# A Consumer of Pins

**Published:** 1899-09

**Authors:** 


### A CONSUMER OF PINS.

Dr. Thomas Annadale reports the case of a twelve-year-old, feeble-minded boy who claimed that he had swallowed a large number of pins. He was, however, not believed, because aside from slight epigastric pain, he neither complained of nor manifested other noteworthy symptoms. Nevertheless, a dose of Castor oil was administered, following which he passed per rectum a quantity of straight and bent pins. Within the next fourteen days the following bodies were discharged *per vias naturales:* Fifty-two pins, one needle, five large nails, with broad heads, three smaller nails, one shirt button, four carpenter's nails, and finally three shoe nails. The case is noteworthy, in that the patient was almost free from symptoms, notwithstanding the large number of foreign bodies he disposed of.—*Dietetic and Hygiene Gazette, September, 1899.*

